# A Screening Platform to Identify and Tailor Biocompatible Small‐Molecule Catalysts

**DOI:** 10.1002/chem.201904808

**Published:** 2019-11-18

**Authors:** Rudy Rubini, Ilya Ivanov, Clemens Mayer

**Affiliations:** ^1^ Stratingh Institute for Chemistry University of Groningen Nijenborgh 4 9474 AG Groningen The Netherlands

**Keywords:** biocompatible catalysis, catalyst screening, metabolism, non-canonical amino acids, uncaging

## Abstract

Interfacing biocompatible, small‐molecule catalysis with cellular metabolism promises a straightforward introduction of new function into organisms without the need for genetic manipulation. However, identifying and optimizing synthetic catalysts that perform new‐to‐nature transformations under conditions that support life is a cumbersome task. To enable the rapid discovery and fine‐tuning of biocompatible catalysts, we describe a 96‐well screening platform that couples the activity of synthetic catalysts to yield non‐canonical amino acids from appropriate precursors with the subsequent incorporation of these nonstandard building blocks into GFP (quantifiable readout). Critically, this strategy does not only provide a common readout (fluorescence) for different reaction/catalyst combinations, but also informs on the organism's fitness, as stop codon suppression relies on all steps of the central dogma of molecular biology. To showcase our approach, we have applied it to the evaluation and optimization of transition‐metal‐catalyzed deprotection reactions.

## Graphical abstract

**A multi‐well screen** that rapidly reports the catalytic activity of transition‐metal catalysts in presence of live cells is reported. The activity of a catalyst to yield a non‐canonical amino acid (ncAA) from an appropriate precursor is coupled to the incorporation of the nonstandard building block into GFP (quantifiable readout; see scheme).

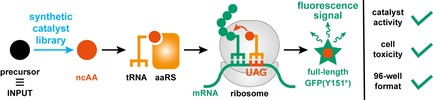

Synthetic chemists and metabolic engineers pursue contrasting approaches to make molecules.^1^ While the former skillfully employ synthetic catalysts and reagents to build up complex molecules, the latter harness the reactivity of biocatalysts in living organisms to produce compounds from fermentation.^2^, ^3^ Although these approaches have been traditionally considered to be incompatible, small‐molecule catalysts that can interface with cellular metabolism have the potential to expand biological function without the need for genetic manipulation.^4^, ^5^, ^6^ For example, such biocompatible catalysts could be part of cellular factories, in which they perform new‐to‐nature transformations to diversify molecules produced by an organism.^7^, ^8^, ^9^, ^10^ Thus, such a concerted effort of synthetic chemistry and metabolic engineering could pave the way toward the direct synthesis of value‐added compounds in cellular settings. Additionally, biocompatible catalysis holds promise for biomedical applications, such as targeted drug release/synthesis,^11^, ^12^, ^13^, ^14^, ^15^ the disruption of cell–cell communication or rescuing dysfunctional enzymes involved in human diseases.^16^

To enable such developments, biocompatible catalysts have to perform a difficult balancing act and function under conditions that both support life and allow an abiological transformations to proceed. This task is complicated by the fact that synthetic chemists routinely perform reactions in organic solvents at temperature and pH regimes that are incompatible with living organisms. Moreover, metabolite concentrations are typically low (<1 mm), compared to the standard substrate concentrations employed in organic synthesis.^5^, ^6^, ^16^ Conversely, the complex intra and extracellular environments of organisms contain a myriad of compounds that can poison exogenously supplied catalysts or reagents.^17^

Consequently, the discovery and optimization of biocompatible catalysts and reactions remain challenging. Typically, a set of potential catalysts is first evaluated for a model transformation under “biologically relevant conditions” (i.e., in presence of water, air and/or thiols) and promising candidates are subsequently tested in biological settings.^12^, ^18^, ^19^, ^20^ The initial evaluation, however, takes neither catalyst/reagent toxicity nor catalyst poisoning by the organism into account. More recently, evaluating biocompatible transition‐metal complexes has also been attempted directly in biological settings by making use of surrogate substrates that either become fluorescent^13^, ^21^, ^22^ or are converted to luciferins^23^ upon a successful transformation. Unfortunately, the observable phenotypes in these screens do not depend on a cellular process and therefore, do not account for an organism's fitness. To address these challenges and further streamline discovery and optimization efforts for biocompatible catalysts, herein, we describe a 96‐well screening platform that rapidly reports on both the activity of a catalyst and the fitness of the organism.

Inspired by the use of genetic circuitry for the directed evolution of enzymes,^24^, ^25^, ^26^ we reasoned that a rapid evaluation and optimization of biocompatible catalysts requires a direct link between a catalyst's activity and an observable phenotype that can only arise in living organisms. Although replacing enzymatic transformations in metabolic pathways with synthetic ones is a possibility to establish such a link,^7^ this strategy is not general and would require genetic knock‐outs that are prone to false positives/negatives. Instead, we envisioned to introduce a simple pathway that 1) is dependent on metabolism while not affecting viability itself; 2) can readily be employed for different reaction types; and 3) can function in organisms ranging from bacteria to mammalian cell lines.

A process that matches these criteria is the site‐specific incorporation of non‐canonical amino acids (ncAAs) into proteins of interest through the suppression of a stop codon by the action of an orthogonal translation system (OTS).^27^, ^28^, ^29^ To repurpose such OTSs for the evaluation and fine‐tuning of biocompatible catalysts, we surmised that the activity of small‐molecule catalysts to give ncAAs from appropriate precursors could be coupled with the subsequent incorporation of these artificial building blocks into green fluorescent protein (GFP) variants (Figure 1 A). Based on these considerations, we constructed a screening platform that comprises three main components: 1) the exogenously supplied catalyst and ncAA precursor (input); 2) an OTS specific for the chemically synthesized ncAA (sensor); and 3) a GFP variant featuring an in‐frame stop codon (reporter). Critically, only upon suppression of the in‐frame stop codon (UAG) full length GFP is produced. Thus, the fluorescence signal detected should relate to ncAA production levels and, as a result, report catalyst proficiency. Moreover, ncAA incorporation relies on all the steps of the central dogma of molecular biology, and therefore should also report on the fitness of the organism (*Escherichia coli* in this study).

**Figure 1:**
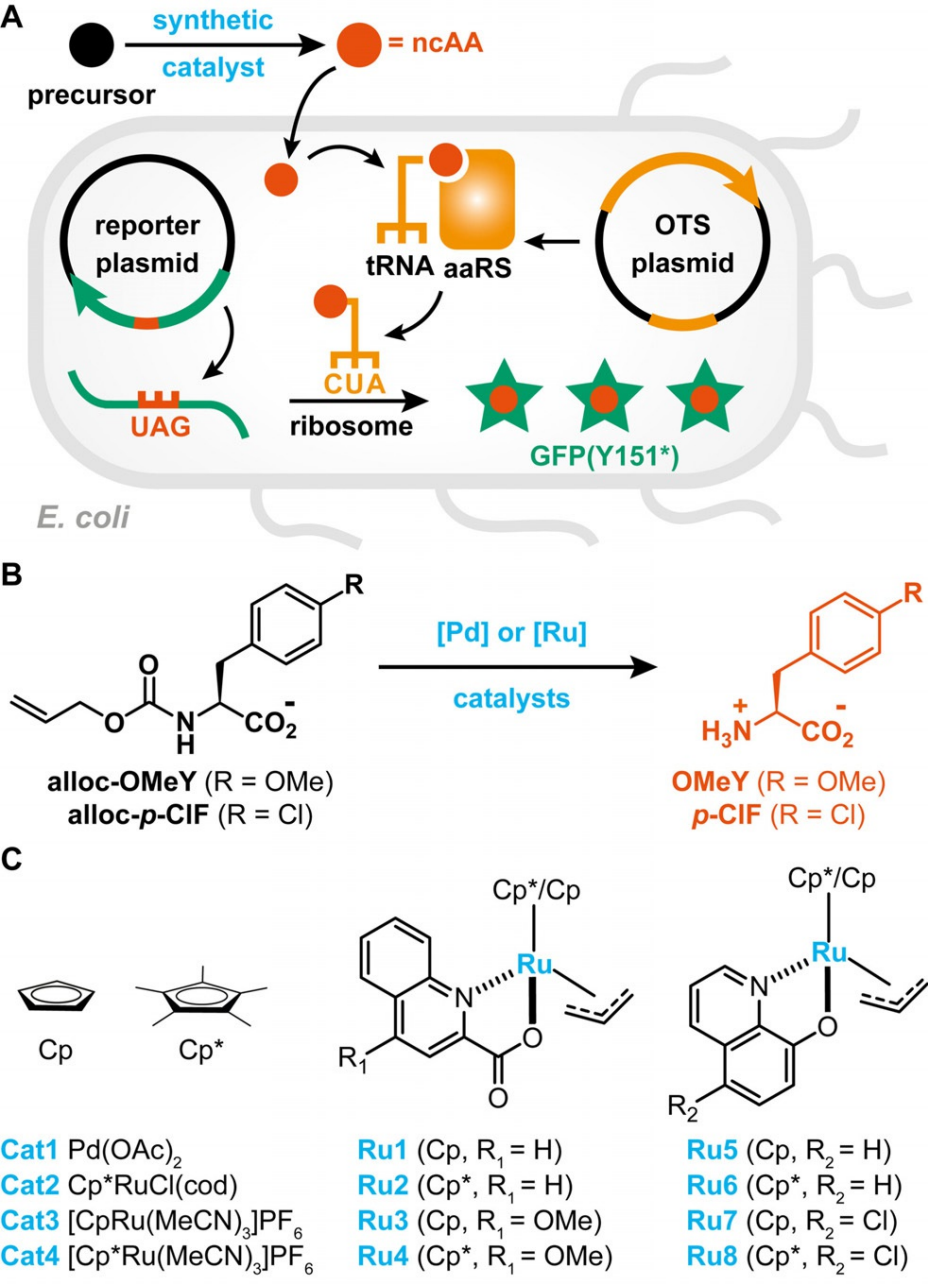
A) Schematic representation of the envisioned screening platform. An appropriate precursor (black sphere) is converted by a synthetic catalyst to a ncAA (red sphere). Note that this transformation can occur outside or inside *E. coli* cells. The synthesized ncAA will then be loaded onto an orthogonal tRNA by an engineered aminoacyl‐tRNA synthetase and incorporated by the ribosome into a GFP variant that features an in‐frame stop codon. B) Uncaging of alloc‐protected ncAAs by palladium or ruthenium catalysts is used as a model reaction in this study. C) Structures of complexes used in this study.

To evaluate the feasibility of the proposed screening platform, we identified the uncaging of allyloxycarbonyl (alloc)‐protected amines as a model reaction from the collection of bioorthogonal/biocompatible transformations reported in the literature.^6^, ^30^ Specifically, this transformation is often employed for the unmasking of prodrugs in vivo^13^, ^31^, ^32^ and can readily be adapted to our proposed screening platform by alloc‐protection of the amine functionality of a ncAA (Figure 1 B). A number of different catalysts have been shown to catalyze this transformation with varying efficiencies. Herein, we selected a set of 12 catalysts (Figure 1 C): four commercially available ones for which low activities were reported (Cat1‐4)^33^, ^34^, ^35^, ^36^ and a total of eight ruthenium‐based half‐sandwich complexes featuring either a quinoline‐2‐carboxylate (Ru1‐4)^18^ or a 8‐hydroxyquinolinate (Ru5‐8)^19^ as bidentate ligand, with some of them showing improved activities when compared to Cat1‐4.

Before evaluating these catalysts in presence of live *E. coli*, we aimed to verify that suppression of an in‐frame stop codon in GFP by an OTS is both a reliable and quantifiable readout. For this, *E. coli* was transformed with two plasmids that encode 1) an OTS based on the promiscuous aminoacyl‐tRNA synthetase, *p*CNF‐RS;^37^ and 2) a GFP variant featuring a UAG stop codon (either Y151* or Y182*, see the Supporting Information for details). Next, we monitored GFP fluorescence in 96 well‐plates after induction of gene expression in LB media containing different concentrations of *p*‐chlorophenylalanine (*p*‐ClF) or *O*‐methyltyrosine (OMeY) over a period of ten hours. As was anticipated, GFP production was dependent on the concentration of the ncAA (Figure 2 A and Figure S1 in the Supporting Information). Plotting the relative fluorescence increase after 200 minutes for the different concentrations yielded a linear correlation, independent of which ncAA and GFP variant was used (Figure 2 B and Figure S2 in the Supporting Information). In contrast, addition of alloc‐protected ncAAs did not result in a significant increase in GFP fluorescence, ensuring a good signal‐to‐noise ratio over two orders of magnitude (10–1000 μm).

**Figure 2:**
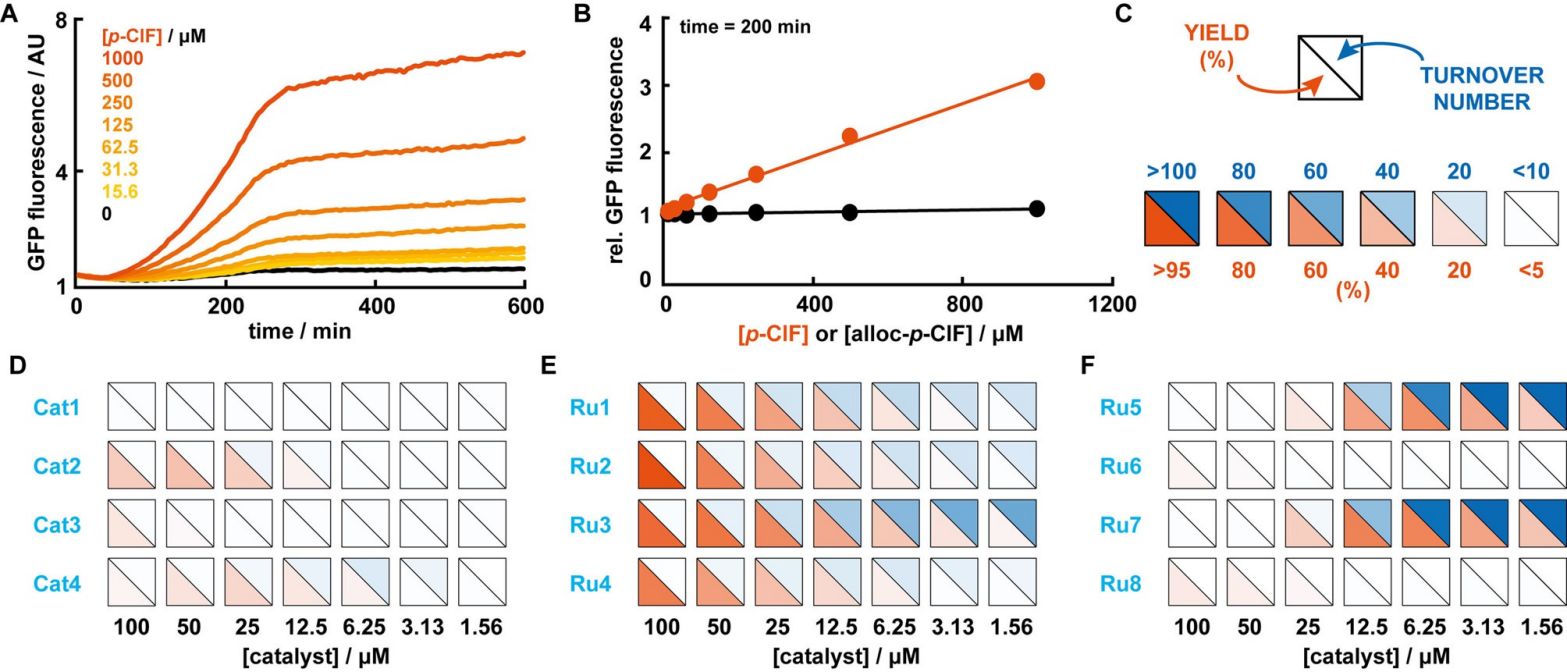
A) Fluorescence (*λ*_ex_=485 nm, *λ*_em_=528 nm) measured over time at different concentrations of *p*‐ClF (concentrations refer to the racemic mixture of d/l *‐p*‐ClF) with sfGFP Y151*. B) Relative increase in fluorescence after 200 minutes for varying concentrations of *p*‐ClF and alloc*‐p*‐ClF (concentrations refer to the respective racemic mixtures). C) Legend depicting representative values for yields and turnover numbers (TONs) for deprotection with transition‐metal complexes. Bright red depicts high yields while bright blue depicts high TONs. D–F) Yields and TONs for the 12 complexes studied at decreasing concentrations. Colors represent the average of at least three independent measurements (see Table S1 the Supporting Information for averages and standard deviations).

To evaluate the proficiencies of the selected transition‐metal complexes to catalyze the deprotection of alloc‐*p*‐ClF, we added decreasing concentrations of each catalyst to the ncAA precursor (1 mm as a racemate) at the time of induction. The 96‐well setup of the screening platform enabled the evaluation of 88 different combinations (+8 samples with varying concentrations of *p*‐ClF for the calibration) in less than four hours. We used the relative increase in GFP fluorescence after 200 minutes to estimate the yields and turnover numbers (TONs) of a catalyst at a given concentration (Figure 2 C). Consistent with their low levels of activity in previous reports,^33^, ^36^ the commercial catalysts (Cat1‐4, Figure 2 D) displayed at best moderate yields (≈35 % in presence of 50 μm Cat2) and low TONs (≈18 for 6.25 μm Cat4, Table S1 in the Supporting Information). Conversely, the more efficient catalysts Ru1‐8 gave rise to higher conversions and TONs (Figures 2 E–F). For example, quantitative conversion (>95 %) was observed at high concentrations (≥100 μm) for the quinoline‐2‐carboxylate bearing complexes, Ru1‐4, with Ru3 also giving rise to >60 turnovers (Figure 2 E and Table S1 in the Supporting Information). Although Cp‐containing complexes Ru1 and Ru3 outperformed the corresponding Cp* derivatives, this difference was more pronounced for the related 8‐hydroxyquinolinate‐ligated complexes, Ru5‐8, for which only Cp complexes Ru5 and Ru7 displayed good activities (Figure 2 F). Another distinct feature of these two complexes was a lack of activity at concentrations >25 μm. Consistent with lower OD_600_ values for high catalyst loadings after the reaction, this observation presumably reflects toxicity of Ru5 and Ru7 at high concentrations rather than a lack of activity. Consequently, the highest yields (>65 %) for Ru5 and Ru7 were observed at a concentration of 12.5 μm. Notably, at low concentrations both catalysts were able to perform approximately 200 turnovers in LB media and in presence of live *E. coli* cells (Table S1 in the Supporting Information).

To confirm that the fluorescence signal results from the site‐specific incorporation of the in situ synthesized *p*‐ClF, we purified GFP variants after performing the deprotection reaction of alloc‐*p*‐ClF with Ru3 (50 μm) and Ru7 (12.5 μm) in 100 mL *E. coli* cultures (see the Supporting Information for details). Only in presence of either transition‐metal catalyst, the addition of the ncAA precursor resulted in production of full‐length GFP (as was judged by SDS‐PAGE), with UPLC/MS analysis confirming the successful incorporation of *p*‐ClF (Figure S3 in the Supporting Information).

To independently validate the observed yields/TONs and apparent toxicities for some catalysts, we recovered samples from the screen and 1) quantified the concentration of *p*‐ClF by HPLC; and 2) determined the number of culturable cells on solid media after overnight growth (see the Supporting Information for details). Because neither of these methods lend themselves to the same level of parallelization as the screening platform, the independent validation was restricted to four complexes, Ru3‐5 and Ru7. When comparing yields determined by HPLC with those obtained from relative GFP fluorescence, we observed a good correlation for Ru3 and Ru4 for all concentrations, whereas Ru5 and Ru7 only showed comparable yields at lower concentrations (Figures 3 A–D). As was expected, quantitative conversions measured by HPLC at high concentrations for these two catalysts contrasted those obtained by GFP fluorescence, a readout that takes biocompatibility into account. Further evidence that high concentrations of Ru5 and Ru7 are indeed not biocompatible derives from the fact that we did not observe growth of *E. coli* on solid media following the reaction (Figures 3 E and S4 in the Supporting Information). Although we still observed a significant decrease of culturable cells for Ru5 or Ru7 at concentrations that give rise to the highest yields (12.5 μm), these conditions seem to offer the best compromise between performance and biocompatibility. Notably, neither Ru3 nor Ru4 display any significant toxicity in the tested conditions (Figures 3 E and S4 in the Supporting Information).

**Figure 3:**
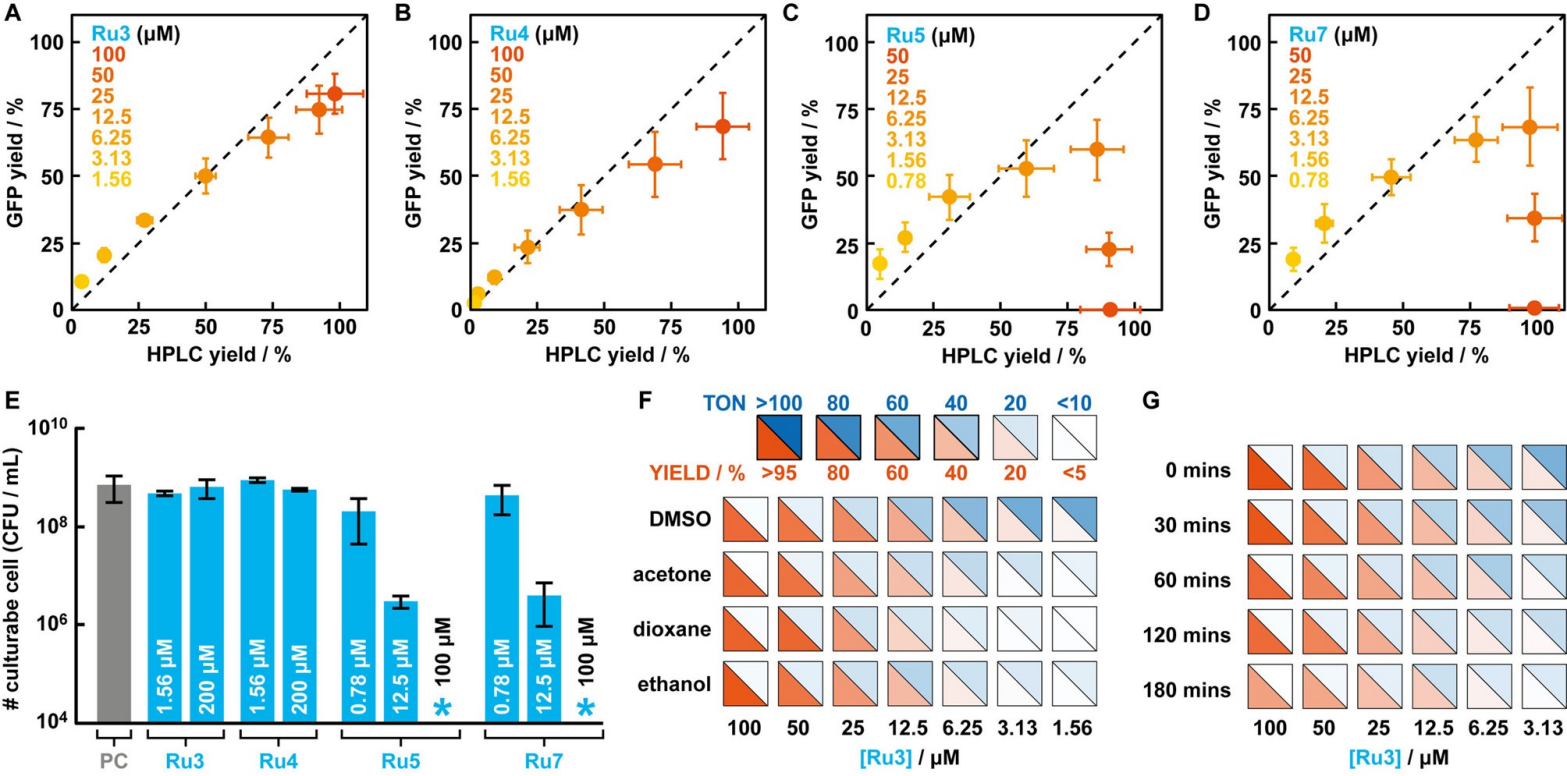
A–D) Comparison of yields determined by GFP fluorescence (*y* scale) and by HPLC quantification (*x* scale) for varying concentrations of Ru3 (A), Ru4 (B), Ru5 (C), and Ru7 (D). The dotted line indicates an ideal correlation between both quantification methods. Data points and error bars are the average yields and standard deviations of at least 3 independent experiments (see also Table S2 the Supporting Information). E) Bar graph showing the number of culturable cells (in colony forming units (CFU) per mL sample) after deprotection of alloc*‐p*‐ClF with varying concentrations of Ru3‐5 and Ru7. Blue stars indicate that no colonies were obtained after overnight incubation. F) Effect on the catalytic performance of varying concentrations of Ru3 in presence of different co‐solvents (all 2.5 % (v/v)). Colors represent the average yields (red) and TONs (blue) of three independent measurements (see Table S3 in the Supporting Information for values and standard deviations). G) Catalytic performance of Ru3 at decreasing concentrations after incubation with growing *E. coli* cells for up to three hours prior to addition of the ncAA precursor. Colors represent the average of three independent measurements (see Table S4 in the Supporting Information for values and standard deviations). Color code same as in Figure 3 F.

Based on its negligible toxicity and good performance, we selected Ru3 to demonstrate that our screening platform could guide the fine tuning of reaction conditions in the future. Anticipating that a biocompatible catalyst needs to perform under varying conditions, we first studied the effect of different co‐solvents on catalyst performance. When comparing yields and TONs in presence of either acetone, dioxane, ethanol, or DMSO (all 2.5 % (v/v)), Ru3 displayed comparable activities in all four co‐solvents at high concentrations, whereas DMSO was the preferred solvent for low catalyst loadings (Figure 3 F and Table S3 in the Supporting Information). Lastly, a biocompatible catalyst should also function in concert with cells and retain its activity over an extended period of time. To determine the extent Ru3 undergoes deactivation in presence of growing *E. coli* cultures, we added the catalyst up to three hours prior to addition of the substrate. Notably, Ru3 proved to be durable and retained >50 % of its initial activity over the three hours period (Figure 3 G and Table S4 in the Supporting Information). Combined, these results augur well that biocompatible catalysts, such as Ru3, can perform in concert with cells under varying conditions and over extended periods of time, and thereby, will find future applications in constructing cellular factories that produce high‐value compounds on demand.

In summary, our work introduces a versatile and operationally simple screening platform to evaluate and optimize biocompatible small‐molecule catalysts. Specifically, the incorporation of an in situ synthesized ncAA into GFP through stop‐codon suppression yields a fluorescence readout that accurately accounts for both the performance and biocompatibility of a catalyst. Moreover, the screen can be performed in 96‐well format, enabling a rapid and straightforward assessment of potential catalysts in parallel in small volumes. As long as the activity of a non‐enzymatic transformation can be linked to the synthesis of a genetically encodable ncAA,^27^, ^28^, ^29^ the platform should readily be applicable to evaluate biocompatible catalysts for other types of transformations. Beyond discovery efforts, we expect that our method will also allow the fine tuning of reaction conditions in a workflow that is akin to method development in organic synthesis. Although we are only at the beginning of seamlessly merging small‐molecule catalysts with cellular metabolism, biocompatible catalysts identified through this screen could ultimately upgrade cellular metabolism and find use in biotechnological or biomedical applications.

## Conflict of interest

The authors declare no conflict of interest.

## Supporting information

As a service to our authors and readers, this journal provides supporting information supplied by the authors. Such materials are peer reviewed and may be re‐organized for online delivery, but are not copy‐edited or typeset. Technical support issues arising from supporting information (other than missing files) should be addressed to the authors.

Supplementary
