# Supplementary material for: A Screening Platform to Identify and Tailor Biocompatible Small‐Molecule Catalysts
Source: Chemistry. 2019 Nov 18;25(70):16017–21. doi: 10.1002/chem.201904808 (PMC6972700; doi:10.1002/chem.201904808)
Supplement: Supplementary file 1 — Supplementary [file CHEM-25-16017-s001.pdf]

CHEMISTRY
A European Journal

Supporting Information

A Screening Platform to Identify and Tailor Biocompatible Small-Molecule Catalysts

Rudy Rubini, Ilya Ivanov, and Clemens Mayer*[a]

chem_201904808_sm_miscellaneous_information.pdf

Table of contents

1. Supporting Figures S2
2. Supporting Tables S4
3. Materials & Methods S6
4. Chemical Synthesis S8
5. Molecular Biology S16
6. Experimental Section S17
7. References S22

1. Supplementary Figures

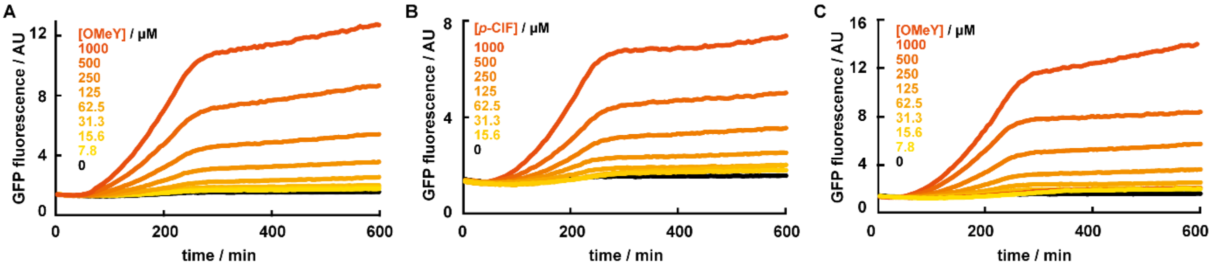

Fig. S1: Fluorescence (λ_{ex} = 485 nm, λ_{em} = 528 nm) measured over time at different concentrations of p-OMeY (**A, C**) or *p*-ClF (**B**, concentrations refer to the racemic mixture of *D/L-p*-ClF) with sfGFP_Y151* (**A**) or sfGFP_Y182* (**B-C**).

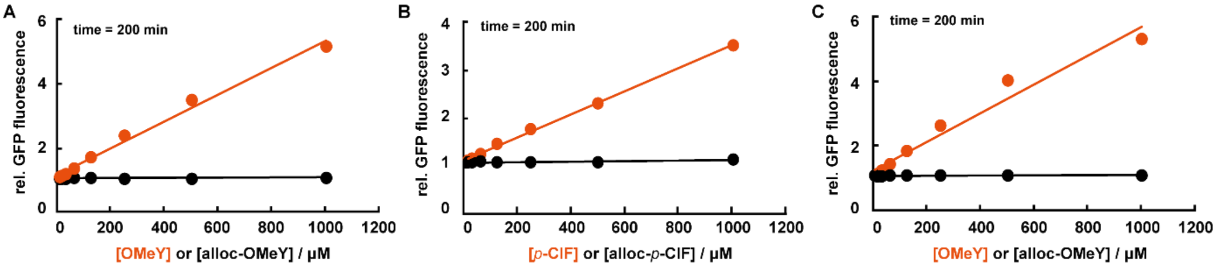

Fig. S2: Relative increase in fluorescence after 200 minutes for varying concentrations of OMeY and alloc-OMeY (**A, C**) or *p*-ClF and alloc-*p*-ClF (**B**, concentrations refer to the respective racemic mixtures) for sfGFP_Y151* (**A**) or sfGFP_Y182* (**B-C**).

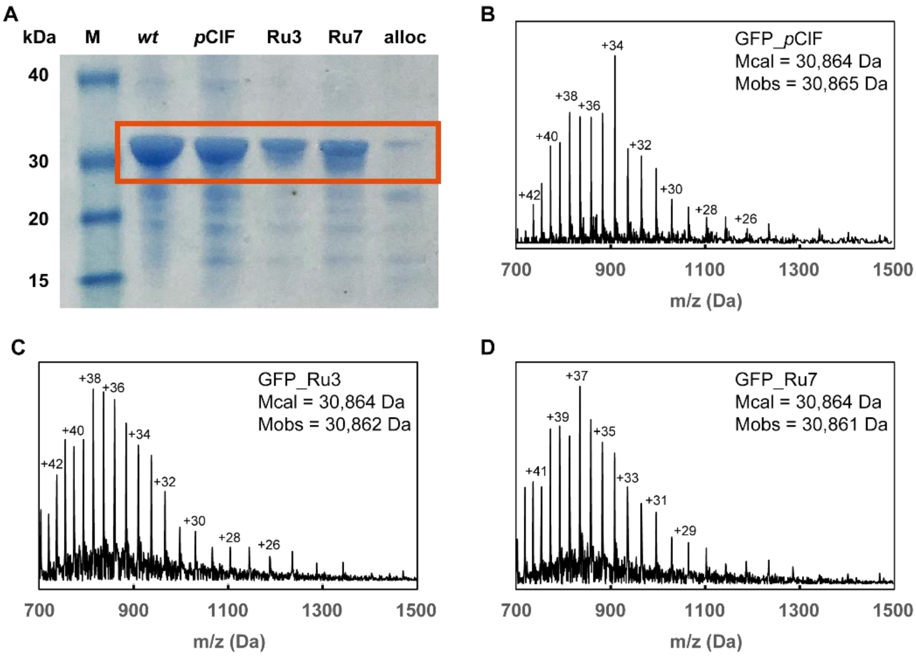

Fig. S3: A: In situ deprotection of alloc-*p*-ClF results in production of full-length GFP_Y151_pClF (red box). *wt* refers to sfGFP, while sfGFP_Y151_*p*-ClF was produced in presence of: pClF = 1 mM pClF, Ru3 = 1 mM alloc-*p*-ClF and 50 µM Ru3, Ru7: 1 mM alloc-*p*-ClF and 12.5 µM Ru7, alloc = 1 mM alloc-*p*-ClF. **B-D:** Mass spectrometry results for GFP variants obtained after purification. *p*ClF, Ru3, and Ru7 refer to the same as before.

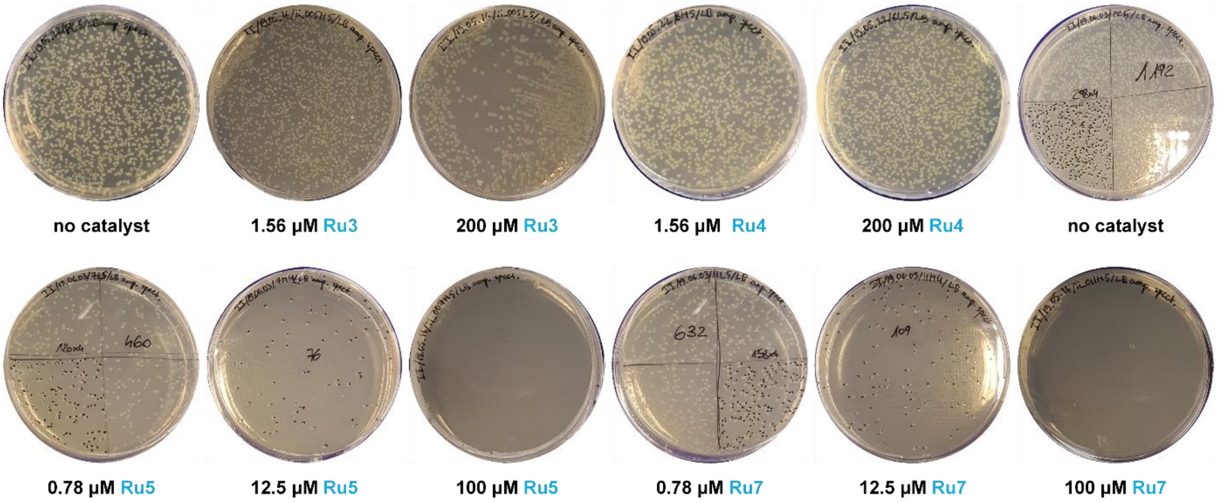

Fig. S4: Representative examples of LB agar plates used to determine the number of culturable cells after deprotection of alloc-*p*-ClF (1 mM) with catalysts Ru3-5 and Ru7 at the given concentration (see **Fig. 3E**). Plates shown are 10^5-fold dilutions of the original sample.

2. Supplementary Tables

Table S1: Yields and TONs for the 12 complexes studied at decreasing concentrations. Numbers represent the averages and standard deviations of at least three independent measurements. Averages were used for **Figs. 2D-F**.

[cat] µM	cat1				cat2				cat3				cat4			
	yield / %		TON		yield / %		TON		yield / %		TON		yield / %		TON	
	avg	stdv	avg	stdv	avg	stdv	avg	stdv	avg	stdv	avg	stdv	avg	stdv	avg	stdv
**100**	0.0	0.0	0.0	0.0	29.7	7.0	3.0	0.1	16.7	0.3	1.7	0.0	9.4	2.4	0.9	0.2
**50**	0.0	0.0	0.0	0.0	35.7	2.9	7.1	0.1	7.8	2.1	1.6	0.4	18.5	0.5	3.7	0.1
**25**	0.0	0.0	0.0	0.0	28.3	4.5	11.3	0.5	1.5	1.4	0.6	0.5	24.3	1.9	9.7	0.8
**12.5**	0.0	0.0	0.0	0.0	10.3	10.0	8.2	1.0	0.4	0.6	0.3	0.4	16.5	3.5	13.2	2.8
**6.25**	0.0	0.0	0.0	0.0	4.0	3.1	6.3	0.3	0.0	0.0	0.0	0.0	11.3	0.3	18.1	0.5
**3.13**	0.0	0.0	0.0	0.0	0.3	2.2	0.9	1.3	0.0	0.0	0.0	0.0	3.8	3.3	12.3	10.7
**1.56**	0.0	0.0	0.0	0.0	0.0	0.0	0.0	0.0	0.0	0.0	0.0	0.0	0.9	1.2	5.6	7.9
	**Ru1**				**Ru2**				**Ru3**				**Ru4**			
**100**	88.3	7,03	8.8	0,70	103.3	6,88	10.3	0,69	81.3	8.8	7.8	1.1	70.9	14.7	6.7	1.5
**50**	71.8	2,93	14.4	0,59	69.4	4,61	13.9	0,92	75.0	10.0	14.3	2.3	54.9	11.5	10.5	2.2
**25**	52.3	6,58	20.9	2,63	46.2	3,11	18.5	1,24	64.0	6.4	24.4	3.5	36.1	8.4	14.0	3.1
**12.5**	34.2	1,07	27.4	0,86	29.0	1,66	23.2	1,33	48.1	6.5	36.9	5.7	23.2	5.8	17.9	4.4
**6.25**	17.7	1,48	28.3	2,37	14.4	1,61	23.0	2,57	32.4	2.8	49.8	5.9	12.1	2.8	19.0	4.0
**3.13**	7.7	0,36	24.7	1,16	6.8	1,88	21.7	6,01	18.7	1.2	57.7	5.7	4.8	2.1	14.6	6.1
**1.56**	3.5	0,47	22.4	3,02	2.9	1,27	18.6	8,11	9.7	2.3	60.7	13.0	1.9	1.4	11.0	8.4
	**Ru5**				**Ru6**				**Ru7**				**Ru8**			
**100**	0.0	0.0	0.0	0.0	9.6	0,10	1.0	0,01	0.0	0.0	0.0	0.0	15.3	1,28	1.5	0,13
**50**	0.2	0.3	0.0	0.1	6.8	0,09	1.4	0,02	0.1	0.0	0.2	0.0	12.7	1,18	2.5	0,24
**25**	16.4	12.7	5.7	4.8	3.4	0,96	1.4	0,38	29.3	9.6	6.0	5.3	9.0	0,88	3.6	0,35
**12.5**	51.2	17.5	35.7	16.8	0.3	0,38	0.2	0,30	68.1	46.7	12.3	19.4	3.5	0,30	2.8	0,24
**6.25**	61.0	14.5	84.2	36.0	0.0	0,00	0.0	0,00	67.4	94.6	11.8	34.0	1.7	0,86	2.8	1,37
**3.13**	48.4	13.8	134.1	60.1	0.0	0,00	0.0	0,00	50.9	143.3	7.9	48.9	0.4	0,31	1.2	0,99
**1.56**	30.4	11.5	170.3	83.6	0.0	0,00	0.0	0,00	33.6	192.0	8.0	68.3	1.2	0,94	7.5	6,01

Tables S2: Yields for catalysts Ru3-5 and Ru7 determined from GFP fluorescence (screen) and HPLC quantification. Averages and standard deviations were obtained from at least three independent measurements. Averages were used for **Figs. 3A-D**.

[cat] µM	Ru3				Ru4				Ru5				Ru7			
	yield GFP		yield HPLC		yield GFP		yield HPLC		yield GFP		yield HPLC		yield GFP		yield HPLC	
	avg	stdv	avg	stdv	avg	stdv	avg	stdv	avg	stdv	avg	stdv	avg	stdv	avg	stdv
**100**	81.3	8.8	96.3	12.5	70.9	14.7	90.4	8.4								
**50**	75.0	10.0	90.4	9.6	54.9	11.5	65.5	9.6	0.2	0.3	94.7	11.2	0.1	0.2	98.9	9.2
**25**	64.0	6.4	70.5	6.2	36.1	8.4	38.5	8.2	16.4	12.7	96.9	8.4	29.3	6.0	99.0	10.2
**12.5**	48.1	6.5	48.6	3.6	23.2	5.8	19.2	4.7	51.2	17.5	93.3	9.9	68.1	12.3	97.4	10.3
**6.25**	32.4	2.8	25.8	2.1	12.1	2.8	7.6	2.5	61.0	14.5	66.2	10.1	67.4	11.8	77.1	8.2
**3.13**	18.7	1.2	11.5	2.2	4.8	2.1	1.7	2.9	48.4	13.8	36.0	7.8	50.9	7.9	45.5	7.3
**1.56**	9.7	2.3	3.3	2.2	1.9	1.4	0.8	1.4	30.4	11.5	16.3	1.8	33.6	8.0	20.4	3.0
**0.78**									19.9	10.3	6.8	1.8	18.4	4.7	8.8	2.0

Table S3: Yields and TONs for Ru3 at varying concentrations in different cosolvents. Averages and standard deviations were obtained from at least three independent measurements. Averages were used for **Figs. 3F**.

[cat] µM	Ru3 DMSO				Ru3 acetone				Ru3 dioxane				Ru3 ethanol			
	yield / %		TON		yield / %		TON		yield / %		TON		yield / %		TON	
	avg	stdv	avg	stdv	avg	stdv	avg	stdv	avg	stdv	avg	stdv	avg	stdv	avg	stdv
**100**	81.3	8.8	7.8	1.1	82.7	13.9	6.6	1.4	84.7	4.5	8.5	0.5	91.9	11.7	9.2	1.2
**50**	75.0	10.0	14.3	2.3	76.5	2.4	11.8	0.5	82.7	3.0	16.5	0.6	75.5	7.1	15.1	1.4
**25**	64.0	6.4	24.4	3.5	51.8	3.4	18.0	1.4	57.0	6.5	22.8	2.6	57.3	6.7	22.9	2.7
**12.5**	48.1	6.5	36.9	5.7	36.7	9.0	25.0	7.2	26.0	4.3	20.8	3.5	39.4	15.7	31.5	12.6
**6.25**	32.4	2.8	49.8	5.9	17.2	4.3	28.5	6.8	10.6	2.4	17.0	3.9	14.6	1.6	23.3	2.6
**3.13**	18.7	1.2	57.7	5.7	5.9	0.7	23.2	2.3	3.8	1.9	12.1	6.2	6.2	1.5	19.8	4.9
**1.56**	9.7	2.3	60.7	13.0	1.2	2.3	13.4	14.8	1.3	1.0	8.3	6.4	2.5	1.3	15.7	8.1

Table S4: Yields and TONs for Ru3 at varying concentrations after incubation with live *E. coli* cells for up to 3 hours. Averages and standard deviations were obtained from at least three independent measurements. Averages were used for **Figs. 3G**.

[cat] µM	t=0 min				t = 30 min				t = 60 min			
	yield / %		TON		yield / %		TON		yield / %		TON	
	avg	stdv	avg	stdv	avg	stdv	avg	stdv	avg	stdv	avg	stdv
**100**	99.2	6.1	9.9	0.6	91.8	11.8	9.2	1.2	81.4	5.4	8.1	0.5
**50**	83.6	8.8	16.7	1.8	75.1	4.4	15.0	0.9	66.1	3.1	13.2	0.6
**25**	57.5	11.3	23.0	4.5	57.4	4.2	23.0	1.7	49.4	4.1	19.8	1.7
**12.5**	40.5	5.8	32.4	4.6	39.0	5.3	31.2	4.3	36.6	5.4	29.3	4.3
**6.25**	27.6	6.7	44.1	10.7	25.2	8.6	40.3	13.7	22.7	3.8	36.3	6.0
**3.13**	17.1	7.0	54.6	22.5	12.9	10.0	41.4	32.0	8.5	5.6	27.1	18.0

[cat] µM	t = 120 min				t = 180 min			
	yield / %		TON		yield / %		TON	
	avg	stdv	avg	stdv	avg	stdv	avg	stdv
**100**	80.8	10.8	8.1	1.1	52.5	6.6	5.3	0.7
**50**	67.3	13.8	13.5	2.8	49.0	7.7	9.8	1.5
**25**	45.6	9.3	18.2	3.7	36.4	2.3	14.5	0.9
**12.5**	28.2	5.4	22.6	4.3	30.2	7.3	24.1	5.8
**6.25**	15.3	6.7	24.5	10.7	11.5	10.3	18.5	16.4
**3.13**	8.1	4.4	26.0	14.0	5.8	5.0	18.6	15.9

3. Materials & Methods

Chemicals, including *D/L-p*-ClF (racemic mixture) and *L*-OMeY, were purchased from Sigma Aldrich and used without further purification unless otherwise noted. ^{1}H-NMR and ^{13}C-NMR spectra were recorded on a Bruker 600 MHz in $CDCl_3$, DMSO-*d6* or acetonitrile- *d3*. sfGFP-pBAD was a gift from Michael Davidson & Geoffrey Waldo (Addgene plasmid #54519; http://n2t.net/addgene:54519; RRID:Addgene_54519)[1] and pULTRA-CNF was a gift from Peter Schultz (Addgene plasmid #48215; http://n2t.net/addgene:48215; RRID: Addgene_48215).[2] *Escherichia coli* strains NEB5-alpha and BL21(DE3) (*New England Biolabs*) were used for cloning and expression. Primers were synthesized by *Eurofins MWG Operon* (Germany). Plasmid Purification Kits were obtained from *QIAGEN* (Germany) and DNA sequencing carried out by *GATC-Biotech* (Germany). Phusion polymerase was purchased from *New England Biolabs* and Ni-NTA resin (Ni Sepharose™ 6 Fast Flow) from *GE Healthcare Life Sciences* (Germany). Concentrations of DNA and protein solutions were determined based on the absorption at 260 nm or 280 nm on a Thermo Scientific Nanodrop 2000 UV-Vis spectrophotometer. Theoretical molecular weights of proteins were calculated using the Expasy ProtParam tool (http://web.expasy.org./protparam/). UPLC/MS analysis was performed on Waters Acquity Ultra Performance LC with Acquity TQD detector. Separation of proteins was achieved with an Acquity UPLC Protein BEH C4 1.7 µm 2.1x150 mm column and a linear gradient of 90% to 10% water (0.1% FA) in ACN (0.1% FA) in 15 minutes. Analytical HPLC analysis was performed on a Waters Acquity HPLC class system (Waters) equipped with a PDA detector. All analyses were performed using a reversed-phase HPLC column (XSelect-CSH-C_{18}, 5 µm, 4.5×150 mm; Waters) kept at 40 °C and the sample plate was kept at room temperature. Absorbance was monitored at different wavelengths for the different ncAAs and their N-alloc derivatives (λ = 225 nm and λ = 275 nm for OMeY , λ = 223 nm and λ = 266 nm for *p*-ClF). Samples were separated with a gradient from 5 to 95% acetonitrile

(0.1% TFA) in water (0.1% TFA) at a flow rate of 1.0 mL/min. Protected and de-protected amino acids were quantified according to a calibration curve obtained from samples containing different concentrations of authentic standards.

4. Chemical Synthesis

Synthesis of alloc protected ncAAs.

The ncAA (1 mmol, 1.00 eq) was dissolved in 10 mL THF (30% in water) and a 1 M NaOH solution (4 mL, 4 eq.) added. Next, allyl chloroformate (1 eq.) was added dropwise and the reaction stirred for 2 hours at room temperature. Then THF was removed under reduced pressure and the solution acidified (1 M HCl, pH = 2-3). The resulting suspension was extracted 3 times with ethyl acetate and brine, and the combined organic fractions dried with sodium sulfate. Removing ethyl acetate under reduced pressure yielded alloc-protected ncAAs in good purities and yields.

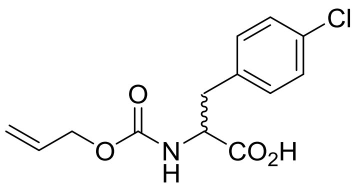

N-alloc- *D/L*-*p*-ClF was obtained as white solid in 69% yield. ^{1}H NMR (600 MHz, Chloroform-*d*) δ 7.21 (d, *J* = 8.0 Hz, 2H), 7.09 – 6.98 (m, 2H), 5.82 (ddt, *J* = 16.4, 10.8, 5.6 Hz, 1H), 5.22 (d, *J* = 17.2 Hz, 1H), 5.18 – 5.13 (m, 1H), 5.09 (d, *J* = 8.2 Hz, 1H), 4.60 (q, *J* = 6.6 Hz, 1H), 4.49 (d, *J* = 5.7 Hz, 3H), 3.12 (dd, *J* = 14.1, 5.5 Hz, 1H), 3.02 – 2.95 (m, 1H). ^{13}C NMR (151 MHz, $CDCl_3$) δ 175.74, 155.70, 133.99, 133.27, 132.33, 130.69 (x2), 128.86 (x2), 118.14, 66.12, 54.40, 37.18. **HRMS** (ESI-positive): *m/z* calculated for $C_{13}H_{14}ClNO_4H$, $[M+H]^+$: 284.06841, found: 284.06871.

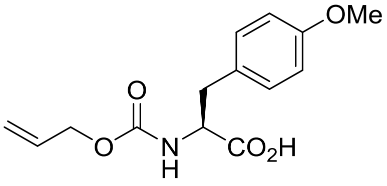

N-alloc-OMeY was obtained as yellow oil in 80% yield.^{1}H NMR (600 MHz, Chloroform-*d*) δ 7.05 – 6.99 (m, 2H), 6.77 (d, *J* = 8.4 Hz, 2H), 5.88 – 5.62 (m, 1H), 5.21 (d, *J* = 17.1 Hz, 1H), 5.17 – 5.09 (m, 1H), 5.07 (d, *J* = 8.2 Hz, 1H), 4.57 (q, *J* = 6.5 Hz, 1H), 4.49 (d, *J* = 5.7 Hz, 2H), 3.72 (s, 3H), 3.11 – 2.84 (m, 2H). ^{13}C NMR (151 MHz, $CDCl_3$) δ 176.13, 158.85, 155.76, 132.47, 130.36 (2x), 127.37, 118.00, 114.17 (2x), 66.01, 55.24, 54.64, 36.91. **HRMS** (ESI-positive): *m/z* calculated for $C_{14}H_{17}NO_5Na$, $[M+Na]^+$: 302.09989, found: 302.10024.

Method A

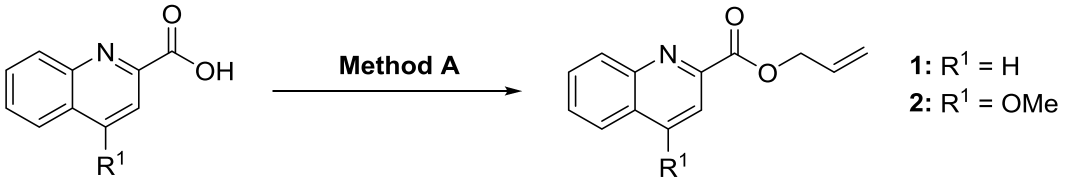

2-quinolinecarboxylic acid (433 mg, 2.5 mmol, 1.0 eq) or 4-methoxy-2-quinolinecarboxylic acid (508 mg, 2.5 mmol, 1.0 eq) and sodium carbonate (424 mg, 4 mmol, 1.6 eq) were dispersed in 10 mL DMF. Allylbromide (622 µL, 3.75 mmol, 1.5 eq) was added dropwise and the suspension stirred overnight at 55 ˚C. The next day, the reaction was quenched by addition of water (50 mL) and the solution extracted three times with dichloromethane (20 mL each). The combined organic phases were extracted twice with brine (25 mL) before drying with sodium sulfate. Dichloromethane was removed under reduced pressure and the crude product subsequently purified by flash chromatography (eluent ethylacetate:hexane: 1:5 → 2:1). Product-containing fractions were pooled and eluents removed to give the ligands **1** and **2**.

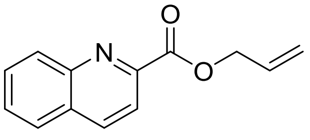

1: Method A gave 492 mg (81% yield) of **1** as colorless oil. ^{1}H NMR (600 MHz, Chloroform-*d*) δ 8.28 – 8.21 (m, 2H), 8.12 (d, *J* = 8.4 Hz, 1H), 7.81 (dd, *J* = 8.2, 1.4 Hz, 1H), 7.72 (ddd, *J* = 8.4, 6.8, 1.4 Hz, 1H), 7.58 (ddd, *J* = 8.1, 6.9, 1.2 Hz, 1H), 6.06 (ddt, *J* = 17.0, 10.2, 5.9 Hz, 1H), 5.41 (dq, *J* = 17.2, 1.5 Hz, 1H), 5.27 (dq, *J* = 10.4, 1.2 Hz, 1H), 4.93 (dt, *J* = 5.9, 1.3 Hz, 2H). ^{13}C NMR (151 MHz, $CDCl_3$) δ 165.10, 148.01, 147.65, 137.28, 131.87, 130.84, 130.26, 129.35, 128.63, 127.53, 121.06, 119.22, 66.78. HRMS: (ESI-positive): m/z calculated for $C_{13}H_{11}NO_2H$, $[M+H]^+$: 214.08626, found: 214.08625.

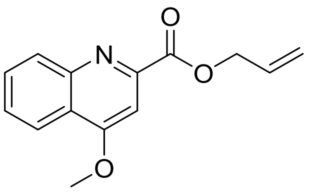

2: Method A gave 394 mg (74 % yield) of **2** as a white solid. ^{1}H NMR (600 MHz, Chloroform-*d*) δ 8.19 – 8.13 (m, 2H), 7.68 (ddd, *J* = 8.5, 6.9, 1.5 Hz, 1H), 7.52 (ddd, *J* = 8.2, 6.9, 1.2 Hz, 1H), 7.51 (s, 1H), 6.06 (ddt, *J* = 17.2, 10.4, 5.9 Hz, 1H), 5.41 (dq, *J* = 17.2, 1.5 Hz, 1H), 5.27 (dq, *J* = 10.4, 1.2 Hz, 1H), 4.92 (dt, *J* = 5.9, 1.3 Hz, 2H), 4.05 (s, 3H). ^{13}C NMR (151 MHz, $CDCl_3$) δ 165.48, 163.34, 149.19, 148.47, 131.91, 130.43, 130.32, 127.60, 122.23, 121.73, 119.29, 100.20, 66.92, 56.08. HRMS: (ESI-positive): m/z calculated for $C_{14}H_{13}NO_3H$, $[M+H]^+$: 244.09682, found: 244.09678.

Method B

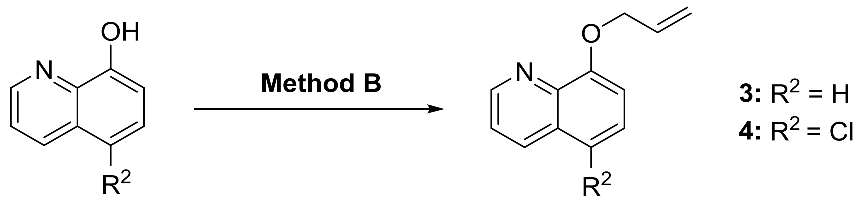

Under a nitrogen atmosphere, 8-hydroxyquinoline (363 mg, 2.5 mmol, 1.0 eq) or 5-chloro-8-quinolinol (449 mg, 2.5 mmol, 1.00 eq) was dissolved in 10 mL DMF. Then, sodium hydride (120 mg, 5 mmol, 2.0 eq) and allylbromide (829 µL, 5 mmol, 2.0 eq) were added. The mixture was stirred overnight at room temperature while stirring. The next day, the reaction was quenched by addition of water (50 mL) and the resulting solution extracted three times with dichloromethane (20 mL each). The combined organic phases were extracted twice with brine (25 mL) before drying with sodium sulfate. Dichloromethane was removed under reduced pressure and the crude product subsequently purified by flash chromatography (eluent ethylacetate:hexane: 1:5 → 2:1). Product-containing fractions were pooled and eluents removed to give the ligands **3** and **4**.

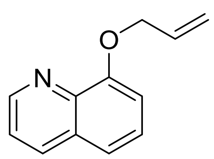

3: Method B gave 332 mg (72 % yield) of **3** as a yellow oil. ^{1}H NMR (600 MHz, Chloroform-*d*) δ 8.88 (dd, *J* = 4.2, 1.8 Hz, 1H), 8.05 (dd, *J* = 8.3, 1.8 Hz, 1H), 7.39 – 7.33 (m, 2H), 7.31 (dd, *J* = 8.2, 1.3 Hz, 1H), 7.00 (dd, *J* = 7.6, 1.3 Hz, 1H), 6.15 (ddt, *J* = 17.3, 10.7, 5.5 Hz, 1H), 5.40 (dq, *J* = 17.3, 1.6 Hz, 1H), 5.26 (dq, *J* = 10.6, 1.4 Hz, 1H), 4.80 (dt, *J* = 5.5, 1.5 Hz, 2H). ^{13}C NMR (151 MHz, $CDCl_3$) δ 154.30, 149.33, 140.39, 135.94, 133.19, 129.51, 126.59, 121.60, 119.73, 118.33, 109.30, 69.84. HRMS: (ESI-positive): *m/z* calculated for $C_{12}H_{11}NOH$, $[M+H]^+$: 186.09134, found: 186.09119.

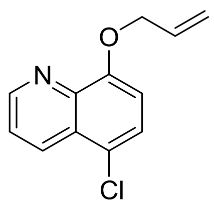

4: Method B gave 374 mg (68 % yield) of **4** as a yellow solid. ^{1}H NMR (600 MHz, Chloroform-*d*) δ 8.93 (dd, *J* = 4.2, 1.7 Hz, 1H), 8.46 (dd, *J* = 8.5, 1.7 Hz, 1H), 7.47 (dd, *J* = 8.5, 4.2 Hz, 1H), 7.44 (d, *J* = 8.4 Hz, 1H), 6.92 (d, *J* = 8.4 Hz, 1H), 6.12 (ddt, *J* = 17.3, 10.7, 5.5 Hz, 1H), 5.40 (dq, *J* = 17.3, 1.6 Hz, 1H), 5.28 (dq, *J* = 10.5, 1.4 Hz, 1H), 4.79 (dt, *J* = 5.4, 1.5 Hz, 2H). ^{13}C NMR (151 MHz, $CDCl_3$) δ 153.51, 149.78, 140.88, 133.04, 132.76, 127.13, 126.35, 122.34, 122.30, 118.64, 109.24, 70.06. HRMS: (ESI-positive): *m/z* calculated for $C_{12}H_{10}ClNOH$, $[M+H]^+$: 220.05237, found: 220.05236.

Method C

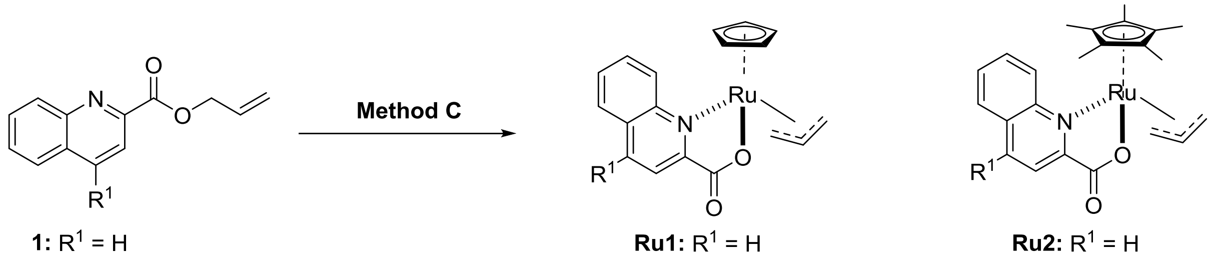

1: R^1 = H
2: R^1 = OMe

Ru1: R^1 = H
Ru3: R^1 = OMe

Ru2: R^1 = H
Ru4: R^1 = OMe

$[CpRu(MeCN)_3]PF_6$ (100 mg, 0.23 mmol, 1.0 eq) or $[Cp^*Ru(MeCN)_3]PF_6$ (75 mg, 0.15 mmol, 1.0 eq) and the corresponding allyl ester (1.3 eq) were added to a dried Schlenk flask under N_2 atmosphere. Dry acetone (3-5 mL) was added to the flask and the reaction was stirred for 15

minutes. Then, the solvent was removed with (1) a pipette if a precipitate formed during the reaction or (2) under reduced pressure, if the no precipitate was formed. The crude product was washed twice with cold acetone (1-2 mL) and residual solvent removed under reduced pressure.

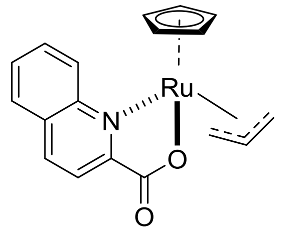

Ru1: Method C gave 27 mg (23% yield) of **Ru1** as yellow solid. ^{1}H NMR (600 MHz, Acetonitrile-d_3) δ 8.81 (d, *J* = 8.3 Hz, 1H), 8.24 (dd, *J* = 8.2, 1.5 Hz, 1H), 8.17 – 8.10 (m, 2H), 7.96 (ddd, *J* = 8.1, 6.9, 1.0 Hz, 1H), 7.93 – 7.88 (m, 1H), 6.20 (s, 5H), 4.76 – 4.66 (m, 2H), 4.53 – 4.45 (m, 1H), 4.42 – 4.37 (m, 1H), 4.22 – 4.17 (m, 1H). ^{13}C NMR (151 MHz, CD_3CN) δ 172.57, 152.64, 148.40, 144.36, 134.14, 132.33, 130.77, 130.35, 129.18, 124.35, 103.73, 96.70 (5x), 71.45, 65.16. HRMS: (ESI-positive): *m/z* calculated for $C_{18}H_{16}NO_2Ru$, $[M-PF_6]^+$: 380.02191, found: 380.02218.

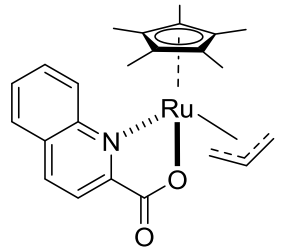

Ru2: Method C gave 19 mg (25% yield) of **Ru2** as light brown solid. ^{1}H NMR (600 MHz, Acetonitrile-d_3) δ 8.77 (d, *J* = 8.3 Hz, 1H), 8.26 – 8.21 (m, 1H), 8.18 (d, *J* = 8.3 Hz, 1H), 8.10 (ddd, *J* = 8.7, 6.9, 1.5 Hz, 1H), 7.94 (ddd, *J* = 8.1, 6.9, 1.0 Hz, 1H), 7.57 – 7.53 (m, 1H), 4.93 (tt, *J* = 10.6, 6.4 Hz, 1H), 4.29 (dd, *J* = 6.4, 2.8 Hz, 1H), 4.17 (dd, *J* = 6.4, 2.8 Hz, 1H), 3.48 (d, *J* = 10.5 Hz, 1H), 3.01 (d, *J* = 10.8 Hz, 1H), 1.65 (s, 15H). ^{13}C NMR (151 MHz, CD_3CN) δ 172.04, 152.81, 145.77, 143.71, 133.04, 132.73, 131.03, 130.36, 128.95, 124.51, 108.91 (x5), 102.67, 77.99, 66.67, 9.55 (5x). HRMS: (ESI-positive): *m/z* calculated for $C_{23}H_{26}NO_2Ru$, $[M-PF_6]^+$: 450.10016, found: 450.10043.

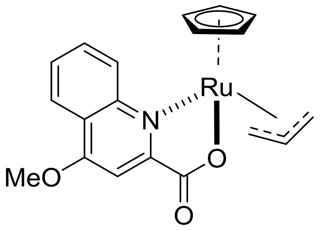

Ru3: Method C gave 71 mg (56% yield) of **Ru3** as yellow solid. ^{1}H NMR (600 MHz, Acetonitrile-d_3) δ 8.44 (ddd, *J* = 8.4, 1.6, 0.6 Hz, 1H), 8.08 (ddd, *J* = 8.7, 6.9, 1.6 Hz, 1H), 7.89 (ddd, *J* = 8.1, 6.9, 1.0 Hz, 1H), 7.80 (dt, *J* = 8.7, 0.8 Hz, 1H), 7.62 (s, 1H), 6.17 (s, 5H), 4.68 – 4.59 (m, 2H), 4.47 – 4.40 (m, 1H), 4.37 – 4.33 (m, 1H), 4.26 (s, 3H), 4.17 – 4.13 (m, 1H). ^{13}C NMR (151 MHz, CD_3CN) δ 172.58, 168.16, 154.11, 148.61, 133.97, 129.37, 128.87, 124.59, 124.12, 104.42, 103.65, 96.57 (5x), 70.64, 65.11, 58.19. HRMS: (ESI-positive): *m/z* calculated for $C_{19}H_{18}NO_3Ru$, $[M-PF_6]^+$: 410.03247, found: 410.03294.

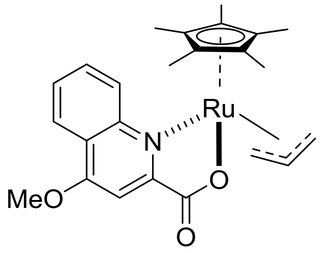

Ru4: Method C gave 56 mg (60% yield) of **Ru4** as dark yellow solid. ^{1}H NMR (400 MHz, Acetonitrile-d_3) δ 8.42 (dd, *J* = 8.4, 1.5 Hz, 1H), 8.03 (ddd, *J* = 8.7, 6.9, 1.5 Hz, 1H), 7.85 (t, *J* = 7.7 Hz, 1H), 7.65 (s, 1H), 7.45 (d, *J* = 8.9 Hz, 1H), 4.87 (ddd, *J* = 10.6, 6.4, 4.2 Hz, 1H), 4.31 – 4.20 (m, 4H), 4.11 (dd, *J* = 6.4, 2.8 Hz, 1H), 3.42 (d, *J* = 10.4 Hz, 1H), 2.98 (d, *J* = 10.7 Hz, 1H), 1.62 (s, 15H). ^{13}C NMR (151 MHz, DMSO) δ 170.71, 166.35, 152.92, 144.69, 131.48, 127.97, 127.29, 123.63, 123.02, 107.32 (5x), 103.27, 101.15, 75.79, 65.26, 56.68, 8.12 (5x). HRMS: (ESI-positive): *m/z* calculated for $C_{24}H_{28}NO_3Ru$, $[M-PF_6]^+$: 480.11072 found: 480.11087

Method D

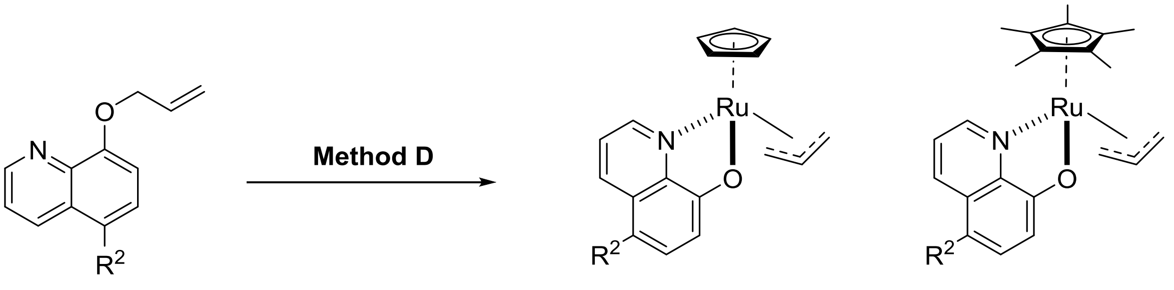

3: R^2 = H
4: R^2 = Cl

Ru5: R^2 = H
Ru7: R^2 = Cl

Ru6: R^2 = H
Ru8: R^2 = Cl

$[CpRu(MeCN)_3]PF_6$ (100 mg, 0.230 mmol, 1.0 eq) or $[Cp^*Ru(MeCN)_3]PF_6$ (75 mg, 0.15 mmol, 1.0 eq) and the corresponding allyl ether (1.0 eq) were added to a dried Schlenk flask under N_2 atmosphere. Dry dichloromethane (3-5 mL) was added to the flask and the reaction was stirred for 15 minutes. Then, the solvent was removed with (1) a pipette if a precipitate formed during the reaction or (2) under reduced pressure, if the no precipitate was formed. The crude product was washed 3 times with 1-2 mL dichloromethane (if a precipitate formed), ethyl acetate or diethyl ether (if the product was soluble in both dichloromethane and ethylacetate). Finally, any residual solvent was removed under reduced pressure.

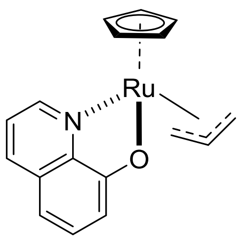

Ru5: Method D gave 28 mg (25% yield) of **Ru5** as reddish-brown solid. ^{1}H NMR (600 MHz, Acetonitrile-d_3) δ 8.63 (dd, *J* = 5.2, 1.2 Hz, 1H), 8.42 (dd, *J* = 8.4, 1.2 Hz, 1H), 7.53 (dd, *J* = 8.4, 5.2 Hz, 1H), 7.41 (t, *J* = 8.0 Hz, 1H), 7.04 (dd, *J* = 8.0, 1.0 Hz, 1H), 6.96 (dd, *J* = 8.0, 1.0 Hz, 1H), 5.97 (s, 5H), 4.56 – 4.40 (m, 2H), 4.17 – 4.08 (m, 3H). ^{13}C NMR (151 MHz, CD_3CN) δ 169.82, 155.80, 146.21, 140.23, 131.35, 130.85, 124.17, 116.13, 112.62, 98.96, 96.26 (x5), 69.36, 62.79. (ESI-positive): *m/z* calculated for $C_{17}H_{16}NORu$, $[M-PF_6]^+$: 352.02699, found: 352.02708.

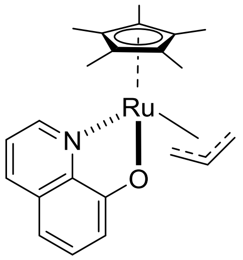

Ru6: Method D gave 56 mg (67% yield) of **Ru6** as light brown solid. ^{1}H NMR (600 MHz, DMSO-d_6) δ 8.52 – 8.46 (m, 2H), 7.62 (dd, *J* = 8.3, 5.1 Hz, 1H), 7.38 (t, *J* = 7.9 Hz, 1H), 7.03 (dd, *J* = 8.0, 1.0 Hz, 1H), 6.97 (dd, *J* = 7.9, 1.0 Hz, 1H), 4.73 (tt, *J* = 10.3, 6.2 Hz, 1H), 4.00 (dd, *J* = 6.1, 2.7 Hz, 1H), 3.87 (dd, *J* = 6.3, 2.7 Hz, 1H), 3.25 (d, *J* = 10.4 Hz, 1H), 2.76 (d, *J* = 10.3 Hz, 1H), 1.56 (s, 15H). ^{13}C NMR (151 MHz, DMSO) δ 167.79, 159.47, 152.88, 145.26, 140.06, 130.61,

124.62, 116.35, 112.24, 107.25 (5x), 97.00, 73.86, 64.67, 8.94 (x5). (ESI-positive): *m/z* calculated for $C_{22}H_{26}NORu$, $[M-PF_6]^+$: 422.10524, found: 422.10502.

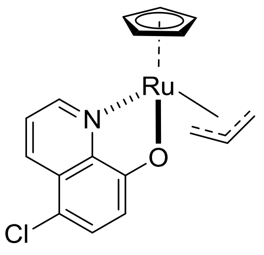

Ru7: Method D gave 90 mg (70% yield) of **Ru7** as red solid. ^1H NMR (600 MHz, Acetonitrile-d_3) δ 8.70 (dd, *J* = 5.1, 1.2 Hz, 1H), 8.63 (dd, *J* = 8.6, 1.2 Hz, 1H), 7.67 (dd, *J* = 8.7, 5.1 Hz, 1H), 7.50 (d, *J* = 8.6 Hz, 1H), 6.91 (d, *J* = 8.5 Hz, 1H), 5.98 (s, 5H), 4.55 (tt, *J* = 10.8, 6.2 Hz, 1H), 4.46 (d, *J* = 10.9 Hz, 1H), 4.20 – 4.10 (m, 3H). ^{13}C NMR (151 MHz, CD_3CN) δ 169.59, 156.43, 146.77, 137.04, 130.36, 128.23, 125.04, 115.64, 113.40, 99.21, 96.37 (x5), 69.71, 63.17. HRMS: (ESI-positive): *m/z* calculated $C_{17}H_{15}ClNORu$, $[M-PF_6]^+$: 385.98802, found: 385.98804.

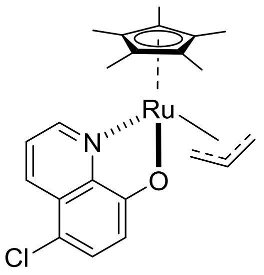

Ru8: Method D gave 38 mg (40% yield) of **Ru8** as yellow solid. ^1H NMR (600 MHz, DMSO-d_6) δ 8.61 – 8.55 (m, 2H), 7.77 (dd, *J* = 8.6, 5.1 Hz, 1H), 7.53 (d, *J* = 8.5 Hz, 1H), 6.95 (d, *J* = 8.5 Hz, 1H), 4.84 (tt, *J* = 10.3, 6.2 Hz, 1H), 4.02 – 4.00 (m, 1H), 3.89 (dd, *J* = 6.4, 2.7 Hz, 1H), 3.28 (d, *J* = 10.4 Hz, 1H), 2.79 (d, *J* = 10.3 Hz, 1H), 1.57 (s, 15H). ^{13}C NMR (151 MHz, DMSO) δ 167.56, 153.63, 145.80, 136.57, 130.23, 127.38, 125.75, 115.92, 112.45, 107.46, 97.34 (x5), 74.28, 65.04, 8.94 (x5). HRMS: (ESI-positive): *m/z* calculated for $C_{22}H_{25}ClNORu$, $[M-PF_6]^+$: 456.06627, found: 456.06589.

5. Molecular Biology

Site-directed mutagenesis: Starting from the previously reported sfGFP-pBAD, the following primers harboring the appropriate mutations were used to generate sfGFP variants with a UAG stop codon in position Y151 or Y182:

Y151TAG_fw:	`CAC AAC GTC TAG ATC ACC GCC GAC AAG CAG`
Y151TAG_rv:	`GC GGT GAT CTA GAC GTT GTG GCT GTT GAA GTT G`
Y182TAG_fw:	`C GAC CAC TAG CAG CAG AAC ACC CCC ATC G`
Y182TAG_rv:	`GTT CTG CTG CTA GTG GTC GGC GAG CTG CAC G`

The following PCR protocol was used: (1) initial denaturation 98 ˚C for 1 min, (2) 16 cycles of denaturation at 98 ˚C for 10 s, annealing at 56 ˚C for 20 s (depending on the Tm of the primers) and extension at 72 ˚C for 2 min 30 s; (3) a final extension at 72 ˚C for 10 min. The resulting PCR product was digested with DpnI for 2 hours at 37 ˚C, purified, and transformed into chemically competent *E. coli* NEB5-alpha cells. A single colony was picked from LB plates containing ampicillin (100 µg/mL) and used to inoculate 5 mL of LB medium containing the same concentration of ampicillin. Bacteria were grown over night, plasmids isolated and variants harboring the correct mutations identified by sequencing. The resulting plasmids sfGFP-pBAD-Y151* or sfGFP-pBAD-Y182* were co-transformed with pULTRA-CNF into chemically-competent *E. coli* BL21(DE3) and a single colony used to inoculate an overnight culture for protein production.

6. Experimental Section

GFP production in 96-well plates in presence of varying concentrations of ncAA: Flasks containing 50 mL Lysogeny broth (LB) medium with 100 µg/mL ampicillin and 50 µg/mL spectinomycin were inoculated with 100 µL of a densely grown overnight culture of *E. coli* BL21(DE3) cells harboring plasmids pULTRA-CNF and sfGFP-pBAD-Y151* or sfGFP-pBAD-Y182*. Cells were incubated at 37 °C and 135 r.p.m. until an optical density at 600 nm of 0.4–0.6 was reached. At this point, gene expression was induced with IPTG (final concentration 1 mM) and *L*-arabinose (final concentration 0.02%). In the meantime, a 96-well plate containing stock solutions of the ncAAs and *N*-alloc-ncAAs dilutions was prepared as follows: OMeY and alloc-OMeY were diluted 1:1 from 10 mM to 78 µM, all in LB medium containing 25% (v/v) DMSO; for *D/L-p*-ClF and *D/L*-alloc-*p*-ClF the concentration of stock solutions was doubled (*i.e.* starting from 20 mM) to have the same concentration of *L-p*-ClF present as for OMeY. Induced *E. coli* cultures and stock solutions were transferred to a new 96-well assay plate (black, clear bottom). Each well contained: (1) 180 µL of induced *E. coli* producing either sfGFP_Y151* or sfGFP_Y182* and (2) 20 µL of either *L*-ncAA or N-alloc-*L*-ncAA dilutions (final concentration range from 1000 µM to 7.8 µM), except the negative controls without ncAAs (here 20 µL of mQ H_2O containing 25% v/v DMSO). Thus, the final volume of each well was 200 µL containing 2.5 % (v/v) DMSO.

At this stage, the assay plate was transferred into a Synergy H1 microplate reader (*BioTek*) that was preheated to 30 °C. Fluorescence intensity ($\lambda_{excitation}$ = 485 nm, $\lambda_{emission}$ = 528 nm) was measured every 5 minutes from the bottom of the wells (7 mm read height) for 16 hours while continuous shaking (double orbital, 425 c.p.m.). The increase in fluorescence over time was calculated as: (current time fluorescence value)/(first reference time fluorescence value), where 30 min is the first reference time value just at the end of the lag phase. The best linear correlation between the relative GFP fluorescence and the concentration of the *L*-ncAAs

was found to be after 200 minutes (end of exponential growth phase) for both OMeY and *D/L*-*p*-ClF incorporation. No significant difference was found comparing the suppression efficiencies at the two positions for the UAG stop codon in sfGFP (**Figs. 2A-B** and **Figs. S1-2**).

Deprotection of alloc- D/L-*p*-ClF in 96-well plates in presence of transition metal complexes: *E. coli* BL21(DE3) cells harboring plasmids pULTRA-CNF and sfGFP-pBAD-Y151* were cultured and induced as described in the previous section. In the meantime a 96-well plate containing stock solutions with varying concentrations of *D/L-p*-ClF, *D/L*-alloc-*p*-ClF and catalysts were prepared as follows: *D/L-p*-ClF was diluted 1:1 from 20 mM to 156 µM, *D/L*-alloc-*p*-ClF were prepared at a fixed concentration of 20 mM in LB media containing 25% (v/v) DMSO. Transition metal complexes were prepared fresh (<30 minutes before beginning to record fluorescence) in H_2O (mQ) containing 25% (v/v) DMSO and diluted 1:1 from 4000 µM to 15.6 µM. After induction, a 96-well (black, clear bottom) assay plate was set up by adding in each well: (1) 180 µL of induced cell culture, (2) 10 µL of either *D/L-p*-ClF dilutions (final concentration ranging from 1000 µM to 7.8 µM) or fixed concentration of *D/L*-alloc-*p*-ClF (final concentration 1000 µM), and (3) 10 µL of either TM complex dilutions (final concentration range from 200 µM to 0.78 µM) or H_2O (mQ) containing 25% (v/v) DMSO as a negative control. Thus, the final volume of each well was 200 µL containing 2.5 % (v/v) DMSO. At this stage, the assay plate was transferred into a Synergy H1 microplate reader (*Biotek*) and fluorescence recorded for 200 minutes as described in the previous section. For each experiment, the samples containing different concentrations of *D/L-p*-ClF were used to obtain a calibration curve and quantify yields and turnover numbers for individual reactions.

HPLC quantification and cell culturability assay: Measurements from the alloc-deprotection in presence of transition metal complexes (see previous section) were stopped after

200 minutes and cells pelleted by centrifugation (3,700 r.p.m. for 5 min, 4 °C). The supernatant (160 μL) was transferred into a fresh 96-well plate containing 2,2'-bipyridine (50 mM in H_2O, 40 μL) to quench the reaction. Selected reaction mixtures were analyzed by HPLC and the concentration of *D/L-p*-ClF and *D/L*-alloc-*p*-ClF determined as described in the Materials & Methods section. Cell pellets from the previous step were quickly resuspended by addition of LB medium (160 μL). Serial dilutions (10^{-5} – 10^{-7}) of selected samples were plated on LB plates containing 100 μg/mL ampicillin and 50 μg/mL spectinomycin and incubated at 37 °C overnight. The following day, colonies were counted to determine the number of culturable cells per mL in the original sample.

Production and purification of sfGFP: Flasks containing 100 mL LB medium with 100 μg/mL ampicillin and 50 μg/mL spectinomycin were inoculated with 100 μL of a densely grown overnight culture of *E. coli* BL21(DE3) cells harboring plasmids pULTRA-CNF and sfGFP-pBAD-Y151*. Cells were incubated at 37 °C and 135 r.p.m. until an optical density at 600 nm of 0.3 was reached. At this point, 1 mM of either *D/L-p*-ClF (as positive control) or *D/L*-alloc-*p*-ClF was added to the cultures. Next, cells were incubated until an optical density at 600 nm of 0.6 was reached and gene expression induced by adding IPTG (final concentration 1 mM) and *L*-arabinose (final concentration 0.02%). At last the transition metal complex was added (50x stock solution in 25% (v/v) DMSO in water, final concentrations: 50 μM for Ru3 and 12.5 μM for Ru7) to the cell mixture containing *D/L*-alloc-*p*-ClF. GFP production was performed for 4 hours at 30 °C while shaking (135 r.p.m.), after which cells were harvested by centrifugation (3,700 r.p.m. for 10 min, 4 °C). Cell pellets were resuspended in buffer (12 mL, 25 mM HEPES, 150 mM NaCl, pH 7.5, containing 1 mg/mL egg white lysozyme and half a tablet of protease inhibitor cocktail (*Roche*)) and incubated for 30 minutes at 4 °C. The cells were then lysed by sonication (7 min, 5 s pulse and pause cycles, 70% amplitude)**,** and cellular

debris was removed by centrifugation (12,000 r.p.m. for 45 min, 4 °C). The supernatant was loaded onto a Ni-NTA resin and purified according to the manufacturer's specifications. Protein-containing fractions were pooled after elution and concentrated. The identity of sfGFP_Y151* and the successful incorporation of *D/L-p*-ClF was confirmed by SDS-PAGE and mass spectrometry (**Fig. S3**).

Co-solvent screening: 96-well plates were set up as previously described in the "Deprotection of alloc-*p*-ClF in 96 well plates in presence of transition metal complexes" section, except *D/L-p*-ClF and *D/L*-alloc-*p*-ClF stock solutions (20 mM) were prepared in LB medium containing 25% (v/v) of the selected co-solvent. *D/L-p*-ClF was then diluted 1:1 from 20 mM to 156 μM as before. The selected transition metal complex (Ru3) was prepared fresh (<30 minutes before beginning to record fluorescence) in H_2O (mQ) containing 25% (v/v) co-solvent and diluted 1:1 from 4000 μM to 15.6 μM. Each stock solution was prepared with one of the following co-solvents: acetone, dioxane, ethanol, DMSO. Finally, the assay plate was transferred into the microplate reader and fluorescence recorded for 200 minutes as previously described. For each experiment, samples containing different concentrations of *D/L-p*-ClF were used to obtain a calibration curve in presence of the specific co-solvent.

Decomposition study: 50 μL of a densely grown overnight culture of *E. coli* BL21(DE3) cells harboring plasmids pULTRA-CNF and sfGFP-pBAD-Y151* were inoculated into 96-deep well plates containing 950 μL LB media and appropriate antibiotics. The resulting 96-deep well plates were incubated at 37 °C while shaking at 900 rpm (Titramax 1000 & Inkubator 1000, Heidolph). Dilutions of the Ru3 (final concentration range from 200 μM to 0.78 μM) were added to the cultures 1, 2, 3, 3.5, and 4 hours after inoculation. Subsequently, protein production was induced by addition of 1 mM IPTG (24 μL of a 50 mM solution) and

0.02% *L*-arabinose (24 μL of a 1% *L*-arabinose solution). *D/L-N*-alloc-*p*-ClF at a final concentration of 1 mM was added to each culture at the time of induction. After that, 200 μL of induced culture were transferred into a 96-well (black, clear bottom) assay plate and the fluorescence measurements were carried out as previously described.

7. References

[1] J. D. Pédelacq, S. Cabantous, T. Tran, T. C. Terwilliger, G. S. Waldo, *Nat. Biotechnol.* **2006**, *24*, 79–88.

[2] K. C. Schultz, L. Supekova, Y. Ryu, J. Xie, R. Perera, P. G. Schultz, *J. Am. Chem. Soc.* **2006**, *128*, 13984–13985.
